# Innovative IntraValvular Impedance Sensing Applied to Biological Heart Valve Prostheses: Design and In Vitro Evaluation

**DOI:** 10.3390/s22218297

**Published:** 2022-10-29

**Authors:** Camilla Gironi, Laura Cercenelli, Barbara Bortolani, Nicolas Emiliani, Lorenzo Tartarini, Emanuela Marcelli

**Affiliations:** eDIMES Lab–Laboratory of Bioengineering, Department of Experimental, Diagnostic and Specialty Medicine (DIMES), University of Bologna, 40138 Bologna, Italy

**Keywords:** heart valve prosthesis, biological heart valve prosthesis, valve thrombosis, electric impedance, implantable sensor, pulse duplicator, continuous monitoring

## Abstract

Subclinical valve thrombosis in heart valve prostheses is characterized by the progressive reduction in leaflet motion detectable with advanced imaging diagnostics. However, without routine imaging surveillance, this subclinical thrombosis may be underdiagnosed. We recently proposed the novel concept of a sensorized heart valve prosthesis based on electrical impedance measurement (IntraValvular Impedance, IVI) using miniaturized electrodes embedded in the valve structure to generate a local electric field that is altered by the cyclic movement of the leaflets. In this study, we investigated the feasibility of the novel IVI-sensing concept applied to biological heart valves (BHVs). Three proof-of-concept prototypes of sensorized BHVs were assembled with different size, geometry and positioning of the electrodes to identify the optimal IVI-measurement configuration. Each prototype was tested in vitro on a hydrodynamic heart valve assessment platform. IVI signal was closely related to the electrodes’ positioning in the valve structure and showed greater sensitivity in the prototype with small electrodes embedded in the valve commissures. The novel concept of IVI sensing is feasible on BHVs and has great potential for monitoring the valve condition after implant, allowing for early detection of subclinical valve thrombosis and timely selection of an appropriate anticoagulation therapy.

## 1. Introduction

Heart Valve Diseases (HVDs) affect more than 100 million people worldwide with a global prevalence of around 2.5%, which rises to 13% amongst patients older than 75 years [1]. Valve repair remains the first-choice surgical treatment for severe HVDs when technically feasible, as it preserves the native valve apparatus. Otherwise, HVDs can be addressed by replacing the affected valve with a heart valve prosthesis (HVP) through surgical valve replacement or transcatheter valve replacement [2]. Some factors that allow identifying the most suitable type of treatment are the patient’s cardiac and extra-cardiac features, the individual risk of surgery and the feasibility of the operation [3].

Heart valve prostheses currently used in cardiac surgery are mechanical heart valves (MHVs) or biological heart valves (BHVs). MHVs have greater longevity but are more susceptible to thrombogenic events, while BHVs have better hemodynamic properties but are associated with a higher risk of structural deterioration causing the need for reoperation [4]. Despite the widespread use of such prostheses, and the continuous progress in both the techniques of their implantation and their design, neither the mechanical nor the biological valves are exempt from complications, which remain an important source of morbidity and mortality [5]. Moreover, since any foreign body implanted within the cardiovascular system is potentially thrombogenic, defining an optimal antithrombotic therapy is crucial to prevent both short- and long-term thrombus formation, a condition known as Prosthesis Valve Thrombosis (PVT), which can lead to stroke [2]. PVT clinically manifests with the formation of obstructive thrombus on the valve leaflets, resulting in an increase in the transvalvular gradient and sometimes symptoms of heart failure, with consequent dysfunction of the prosthesis itself [6].

The precursor of PVT, known as Subclinical Leaflet Thrombosis (SLT), has been described by Makkar et al. [7] as the formation of hypoattenuating structures on the prosthetic aortic valve leaflets and the progressive reduction in their movement. After the publication of this study, several authors reported the widespread presence of SLT in patients undergoing valve replacement, with a prevalence between 5% and 40% [6,7,8,9,10,11,12,13,14]. In particular, SLT is characterized by hypoattenuating leaflet thickening (HALT); impaired coaptation of the leaflets; reduced leaflet motion (RLM); decreased or increased effective orifice area (EOA); increased transvalvular gradient; or valve regurgitation, as well as the absence of evident clinical manifestations of the prosthesis malfunctioning or significant changes in hemodynamic parameters [10]. Of note is the fact that these phenomena mainly affect transcatheter BHVs, rather than surgical BHVs, and can be addressed by the assumption of appropriate anticoagulant therapy [3,15]. The natural history of SLT is not yet fully known, nevertheless, HALT and RLM can still develop not only in the first few months following the implantation, but also over an extended period (up to one year). Therefore, serial-imaging follow-up and proper evaluation of risks and benefits of long-term anticoagulation are extremely important [13]. Given the recent widespread use of heart valve prostheses and the high risk of facing SLT beyond the first year following implantation, it is important to identify the risk of SLT in such a way as to be able to plan an adequate anticoagulant therapeutic strategy, or to correct it if the resulting therapy proves inadequate for the patient, to ensure an optimal valve hemodynamics [16].

Imaging plays a central role in evaluating the functionality of HVPs following their implantation [17]. The postoperative follow-up includes periodic monitoring by echocardiographic examinations which, however, do not allow us to identify the presence of SLT, mainly due to the transvalvular pressure gradients which remain within the physiological range [11]. In particular, Transthoracic Echocardiography (TTE) could only identify HALT in the parasternal long and short axis, while Transesophageal Echocardiography (TEE) could detect both HALT and RLM during diastole and systole, respectively, although it represents too invasive an approach to be performed routinely. Currently, 4D Multi-Detector Computed Tomography (4D-MDCT) is the gold-standard diagnostic tool for the accurate evaluation of both the thickness and the mobility of valve leaflets after implantation, and therefore for the diagnosis of SLT [9]. Nevertheless, its routine adoption into clinical practice would expose patients to a high amount of radiation and would introduce logistical constraints for health facilities.

In recent years, some attempts have been made to conceive an implantable device for the continuous monitoring of HVP functionality after implantation, in order to detect SLT early. Vennemann et al. [18] realized a wireless and battery-less implantable blood flow sensor, consisting of a permanent magnet array, for the remote monitoring of HVP functionality based on Faraday’s law of induction. Another proposal comes from Bailoor et al. [19], who presented a computational “proof-of-concept” study which exploits CFD (Computational Fluid Dynamics) analysis and Supervised Learning to determine the best positioning for embedded pressure sensors near the HVP in order to detect RLM.

We have recently conceived an innovative sensorized HVP [20] that may open up important perspectives for a system capable of monitoring the functionality of HVPs after implantation. The novelty of the system arises from applying impedance measurement techniques currently used in implantable cardiac devices to HVPs in order to (a) detect, as early as possible, the potential presence of subclinical thrombotic formations on the HVP that can alter its functioning, and (b) monitor the progressive deterioration of the prosthesis itself. This would allow for the timely selection of an appropriate anticoagulant therapy prior to compromised cardiac function, resulting in a potential reduction in mortality and improvement in patient quality of life. We have named this impedance-based technique IntraValvular Impedance (IVI) sensing, and we have already designed and tested the first proof-of concept prototypes of IVI sensing as applied to MHVs [21].

As the next step, in this study we present the conceptual design of IVI sensing as applied to BHVs. Different solutions for the sensorization of a commercial BHV model are described and tested on a circulatory mock-loop system reproducing both normal dynamics and abnormal functioning of the valve leaflets.

## 2. Materials and Methods

### 2.1. IVI Sensing—The Concept

**Figure 1:**
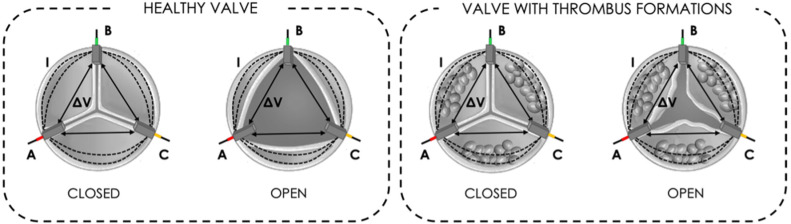
The principle of measurement of IntraValvular Impedance (IVI) for a healthy valve (**left**) and for a valve with thrombus formation (**right**): current pulses (I) are injected between each electrode pair to generate local electric field interference with leaflet movement, and potential difference (∆V) is recorded.

IVI measurement is based on embedding miniaturized electrodes in the structure of the BHV, which are used for the generation of a local electric field by injecting sub-threshold currents (I) between each pair of electrodes, and for the recording of potential differences (∆V) between those same electrodes. Following Ohm’s first law, the impedance measurement (IVI) is obtained as the ratio between the recorded ∆V over the injected I. Since the valve leaflets interfere with the local electric field lines during the valve opening and closing dynamics, IVI variations within the cardiac cycle (ΔIVI) reflect the cyclic movements of the valve leaflets. Thus, it is possible to correlate alterations of the IVI signal with alterations of the mobility of the valve leaflets which may occur in the presence of thrombus formations, as shown in Figure 1.

### 2.2. “Proof-of-Concept” Prototypes

Three “proof-of-concept” sensorized BHVs were assembled and tested. Each prototype was made up of a commercial BHV currently used for surgical aortic valve replacement procedures (Perceval Aortic Valve, LivaNova PCL, London, UK), and was equipped with three electrodes, conventionally called “A”, “B” and “C”. The three electrodes are visually distinguishable from one another based on the color of the heat shrink which covers the electrode–wire interface: red for electrode “A”, green for electrode “B” and yellow for electrode “C”.

The electrodes were manufactured in Pt/Ir, with different sizes and geometries. Pt/Ir is a biocompatible alloy normally used for implantable electrodes thanks to its biocompatibility and corrosion resistance [22]. The positions of the electrodes near the valve leaflets were maintained by sewing them to the BHV structure using suture thread. In addition, a conductor wire has been welded in the longitudinal direction of each electrode’s structure for signal transmission.

The three “proof-of-concept” prototypes provide different geometry, size and positioning of the embedded electrodes in order to determine the optimal configuration that would allow the most sensitive detection of IVI signal. For each pair of electrodes (“AB”, ”BC” and “CA”) an IVI measurement was performed according to the bipolar measurement configuration, which involves the use of a pair of electrodes that simultaneously act as source (for the generation of the local electric field) and receiver (for potential difference recording). A detailed description of each prototype is provided in the following paragraphs.

#### 2.2.1. Prototype 1

**Figure 2:**
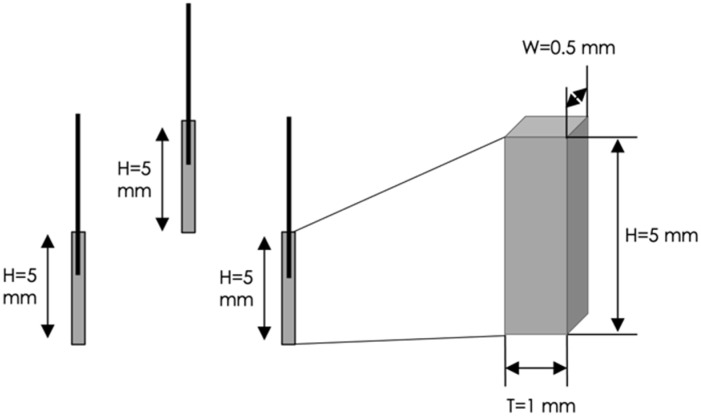
Geometric dimensions of the three electrodes embedded in the Prototype 1.

Prototype 1 involves small parallelepiped-shaped electrodes with the following dimensions: height H = 5 mm, width W = 1 mm and thickness T = 0.5 mm (Figure 2).

**Figure 3:**
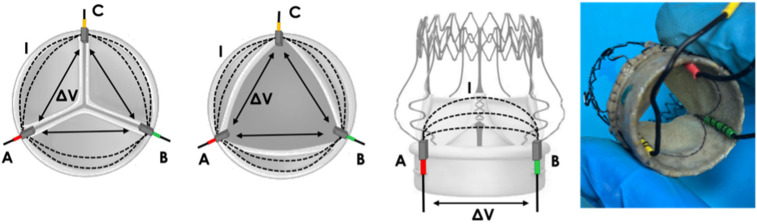
Prototype 1: positioning of “mini-platelet” electrodes A (red), B (green) and C (yellow). Local electric field lines (I) and the corresponding potential difference measurement (ΔV) are represented for each pair of electrodes.

These electrodes, which we called “mini-platelet” electrodes, were positioned in the commissures of the BHV leaflets (Figure 3).

#### 2.2.2. Prototype 2

**Figure 4:**
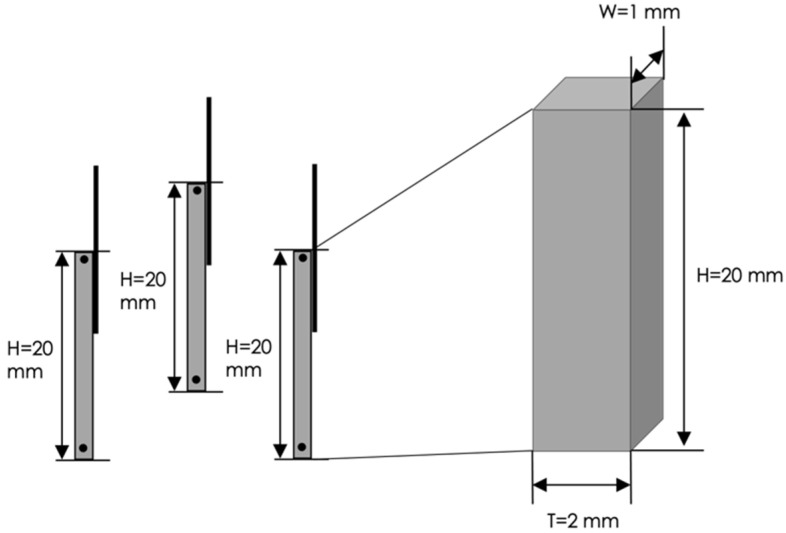
Geometric dimensions of the three electrodes embedded in the Prototype 2.

Prototype 2 involves parallelepiped-shaped electrodes with the following dimensions: height H = 20 mm, width W = 2 mm and thickness T = 1 mm (Figure 4).

**Figure 5:**
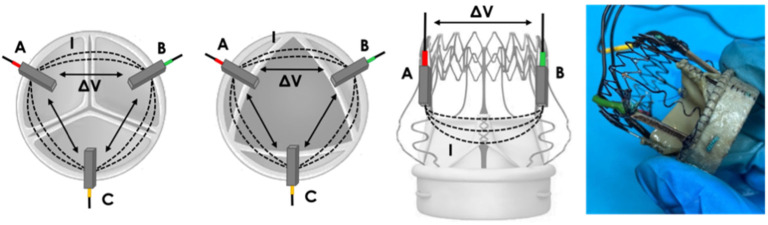
Prototype 2: positioning of “bar” electrodes A (red), B (green) and C (yellow). Local electric field lines (I) and the corresponding potential difference measurement (ΔV) are represented for each pair of electrodes.

These electrodes, which we called “bar” electrodes, were positioned onto the BHV stent along leaflet centerlines (Figure 5).

#### 2.2.3. Prototype 3

**Figure 6:**
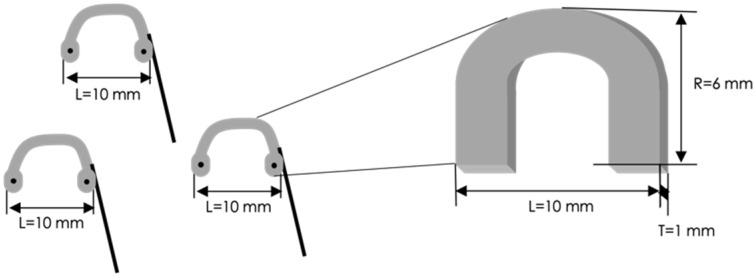
Geometric dimensions of the three electrodes embedded in the Prototype 3.

Prototype 3 involves small arcuated electrodes with the following dimensions: length L = 10 mm, radius R = 6 mm and thickness T = 1 mm (Figure 6).

**Figure 7:**
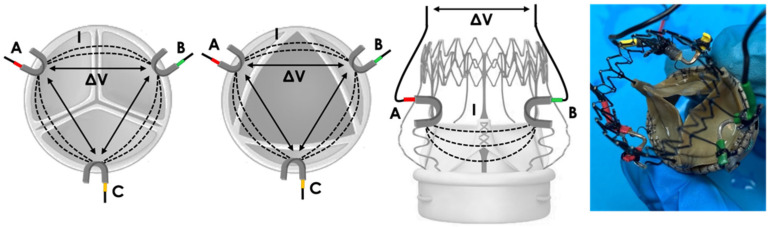
Prototype 3: positioning of “arch” electrodes A (red), B (green) and C (yellow). Local electric field lines (I) and the corresponding potential difference measurement (ΔV) are represented for each pair of electrodes.

These electrodes, which we called “arch” electrodes, were positioned onto the BHV stent along leaflet centerlines (Figure 7).

### 2.3. Dedicated Impedance Measuring Unit

The conductor wires welded to the electrodes for signal transmission were connected to a dedicated external impedance measurement system (“impedance-meter”) which was ad hoc produced by a specialized company (http://www.protechgroup.it/, accessed on 20 October 2022). The impedance-meter is a programmable instrument, i.e., the amplitude of the current excitation can be selected from among 7 values (4.5 μA, 9 μA, 18 μA, 36 μA, 72 μA, 144 μA, and 288 μA) and the pulse frequency from among 5 values (1 KHz, 2 KHz, 4 KHz, 8 KHz, and 12 KHz). These values are within the range of the subthreshold current amplitude and pulse frequency already adopted in impedance sensors typically used for implantable cardiac pacing devices [23,24] and in our previous experience in the field of impedance minute ventilation sensors [25].

The impedance-meter delivers current pulses to each of the three electrodes embedded in the valve prosthesis, thus generating a local electric field near the BHV leaflets. Then, the device records the potential difference between each pair of electrodes ($\Delta V_{AB}$, $\Delta V_{BC}$ and $\Delta V_{CA}$) from which the corresponding IVI measurement is calculated. Hence three IVI signals are obtained for each prototype ($IVI_{AB}$, $IVI_{BC}$ and $IVI_{CA}$).

Taking as reference the Impact Custom Model 2364 (Medtronic, Minneapolis, MN, USA), which was a commercial impedance meter intended to be used with standard cardiac leads, current pulses with 18 μA amplitude and 4 kHz frequency were used in this study.

Furthermore, a calibration procedure (allowed by the fact that the impedance-meter uses known values for internal resistors) was preformed prior each test. This calibration procedure consists of performing an impedance measurement using a known value of internal resistor (1 kOhm). Since the impedance-meter works with the relationship between the measured potential difference and the resistance given by 1 Ohm=2.5 mV, during the calibration procedure it is verified that the read voltage is about 2500 mV, i.e., corresponding to 1 kOhm.

### 2.4. In Vitro Testing

#### 2.4.1. Circulatory Mock-Loop Platform for In Vitro Testing

The sensorized BHV prototypes were tested on a Pulse Duplicator provided by LifeTec Group^TM^ (Eindhoven, The Netherlands), which is a mechanical simulator of the cardiovascular system that allows us to reproduce the systemic circulation and pulsatile behavior of the heart. The Pulse Duplicator was realized by means of a closed-loop hydraulic system (“mock-loop”), based on the Windkessel model. As provided by the Windkessel model, the systemic arterial tree behaves as an elastic reservoir, capable of accumulating blood, which receives pulsatile blood from the left ventricle (through the aortic valve) and supplies blood to the arterioles and capillaries, seen overall as a vascular equivalent to resistance. Accordingly, the assembled mock-loop involves a sensorized BHV prototype in the aortic position, which is in line with the Windkessel module that simulates the vessels downstream the aortic valve. The mock-loop also comprises a gear pump and a reservoir both connected to other components by means of silicon tubes.

Each prototype was inserted into a dedicated housing which was designed and 3D-printed in polymer resin (Form 3 printer, Formlabs, Somerville, MA, USA) in order to not only be compatible with the wiring of the electrodes integrated into the valve but also to allow the bioprosthesis to be kept in position when subjected to the hydrodynamic cycles of opening/closing within the test platform. Each prototype’s housing was then connected to the mock-loop by using an inflow and outflow connector.

The Windkessel module was made of two components both positioned downstream of the aortic BHV: an elastic silicone tube and an occluder. The elastic silicone tube (diameter 25 mm, thickness 1 mm, length 6 cm) represents the systemic aortic tree compliance capable of accumulating the test fluid by expanding itself. The occluder consists of a closed chamber with an adjustable piston which hinders the flow of the test fluid, thus representing the equivalent resistance of peripheral vessels.

**Figure 8:**
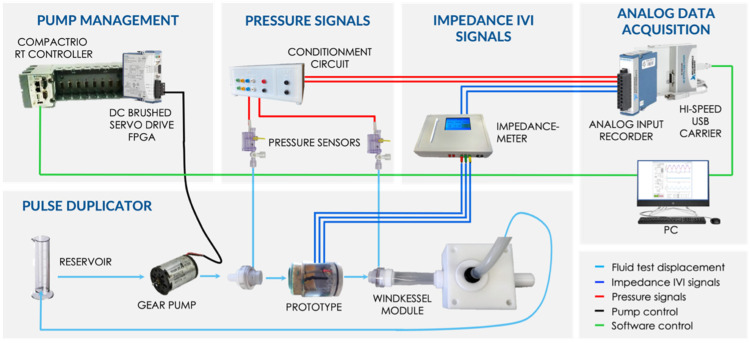
Schematic representation of the circulatory mock-loop platform for in vitro testing.

In addition to the Pulse Duplicator, multiple devices are included in the circulatory mock-loop platform for pump management; for pressure and IVI measurement; and for analog data acquisition (Figure 8).

The pulsatile displacement of the circulatory fluid (NaCl 0.9% in aqueous solution) inside the mock-loop takes place under the action of a gear pump (Maxon Motor AG, Sachseln, Switzerland) controlled by a motion control module (NI9505, cRIO, National Instruments, Austin, TX, USA). The pump controls both the direction and the flow rate of the test fluid, thus generating a pulsatile flow by alternating delivery and suction phases.

Pressure measurement was performed by means of two pressure sensors (Baxter Uniflow, Bentley Laboratories Europe BV, Holland, The Netherlands) positioned upstream and downstream of the BHV, exploiting two dedicated holes on the surface of inflow and outflow connectors, for ventricular and aortic pressure recording, respectively. The two pressure sensors were equipped with a dedicated conditioning circuit for signal amplification and filtering. IVI measurement was performed by connecting the wires, welded to the electrodes, to the impedance-meter.

Both pressure and IVI signals were then transferred to the PC for visualization and recording by means of the Analog Input Recorder module (9201, National Instruments, Austin, TX, USA) and the Hi-Speed USB Carrier module (USB 9162, National Instruments).

#### 2.4.2. Software Interface for Mock-Loop Control and Data Acquisition

**Figure 9:**
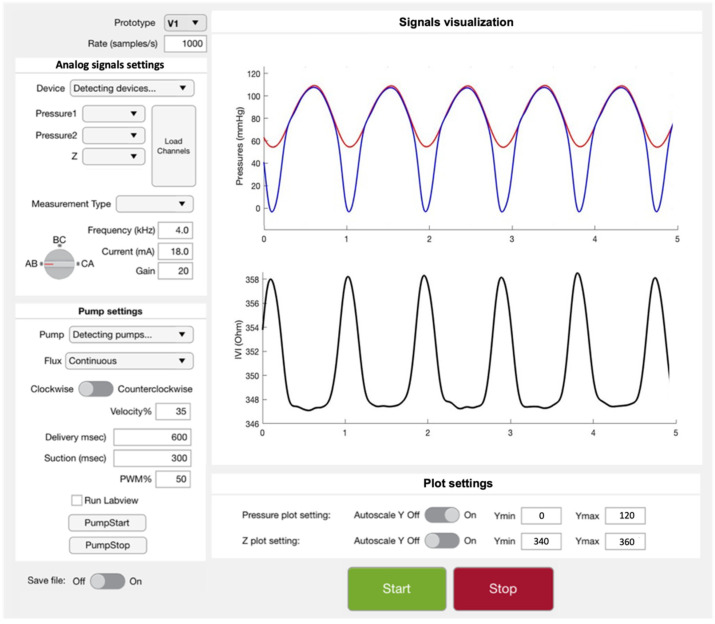
Graphic User Interface (GUI) implemented in MatLab AppDesigner.

A Graphic User-Interface (GUI) was implemented in Matlab App-Designer (R2019a, MathWorks, Natick, MA, USA) to control the circulatory mock-loop platform during in vitro tests. The GUI is divided into the following different sections: one for the settings of the acquired analog signals (“Analog signals settings”), one for driving the pump (“Pump settings”) and one for the real-time visualization of the acquired signals (“Signal visualization”), as shown in Figure 9.

The “Analog signals settings” section allows us to interface the PC with the Analog Input Recorder for pressure and IVI signal acquisition and to set the impedance-meter parameters for IVI measurement.

The “Pump settings” section allows us to set the specifications for the flow displacement inside the mock-loop in terms of flow direction; timing of delivery and suction phases; and PWM (Pulse Wave Modulation, to control the gear pump speed). A second program implemented in LabView (National Instruments, Austin, TX, USA) was run in the background during the tests and allowed us to drive the pump, in accordance with the parameters previously set on the GUI, by using the CompactRIO Real-Time Controller (cRIO 9004, National Instruments) and the DC Brushed Servo Drive module (FPGA 9505, National Instruments), both connected to the PC.

The “Signals visualization” section is dedicated to the visualization of real-time analog signal plots. An additional section at the bottom of the GUI, called “Plot settings”, gives to the user the ability to apply autoscaling to the graphics or to set specific values for axis limits for a better visualization of the acquired signals.

#### 2.4.3. In Vitro Tests with Normal Leaflet Dynamics

A first set of in vitro tests reproduced the “physiological” working condition with the purpose of evaluating the IVI signal during the normal functioning of the valve prosthesis in which the valve leaflets can completely open and close. The valve opening/closing dynamics were obtained by alternating delivery (550 ms) and suction (350 ms) phases. According to the UNI EN ISO 5840 Standard, the mock-loop reproduced the “normotensive” pressure and flow waveforms (i.e., arterial peak systolic pressure 120 mmHg, arterial diastolic pressure 80 mmHg, differential pressure across closed aortic valve equal to 100 mmHg). Each test was conducted under the following environmental conditions: room temperature ~20 °C and relative humidity ~50%. During the tests, the temperature of the working fluid inside the mock-loop platform was maintained at room temperature.

The IVI signal was recorded for each prototype, and IVI variations during the fully opening/closing dynamics of the valve were compared.

#### 2.4.4. In Vitro Tests with Altered Leaflet Dynamics

A second set of in vitro tests reproduced the “altered” working condition with the purpose of evaluating the IVI signal in a hemodynamically altered situation in which the valve leaflets do not fully open and close due to their reduced mobility, induced by a simulated “hypotensive” pressure condition. The valve opening/closing dynamics were obtained by alternating delivery (550 ms) and suction (350 ms) phases. The mock-loop was able to reproduce pressure and flow waveforms (i.e., arterial peak systolic pressure 70 mmHg, arterial diastolic pressure 30 mmHg, differential pressure across closed aortic valve equal to 50 mmHg) similar to the “hypotensive” pressure condition provided by the UNI EN ISO 5840 Standard (i.e., arterial peak systolic pressure 60 mmHg, arterial diastolic pressure 40 mmHg, differential pressure across closed aortic valve equal to 50 mmHg). As before, in this case, each test was also conducted at a room temperature of ~20 °C and relative humidity of ~50%, and with the working fluid temperature maintained at room temperature.

The IVI signal was recorded for each prototype, and IVI variations of the dynamics when the valve was not fully opening/closing were compared.

### 2.5. Data Analysis and Statistics

For each prototype we evaluated the maximum percent variation of the impedance module ($\Delta IVI_{max}\%$) as the ratio between the maximum excursion of IVI measurement when passing from a closed position of the valve ($IVI_{closed}$) to the completely or not-completely open position ($IVI_{open}$), and the IVI measurement corresponding to the closed position, as shown in Equation (1). For each experimental condition, $\Delta IVI_{max}\%$ was reported as Mean Value±SD calculated over 60 cardiac cycles.

(1) $$\Delta IVI_{max}\%=\frac{=\left|IVI_{open}-IVI_{closed}\right|}{IVI_{closed}}*100$$

Statistical analysis was performed using the Student’s *t*-test and allowed a comparative evaluation of both intra-prototype and inter-prototype correlation of IVI measurements. A p-value of 0.05 was chosen as significant. All analyses were made with SPSS version 23.0 (IBM SPSS, New York, NY, USA).

## 3. Results

### 3.1. Results of Tests with Normal Leaflet Dynamics

**Figure 10:**
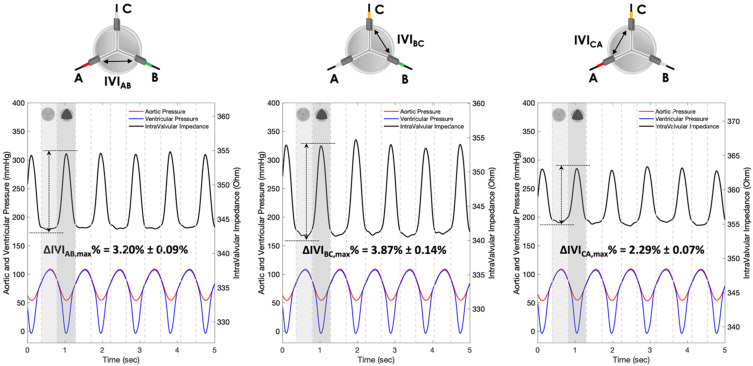
IVI measurements recorded for the three pairs of electrodes of Prototype 1 under normal leaflet dynamics: AB (**left**), BC (**center**) and CA (**right**).

**Figure 11:**
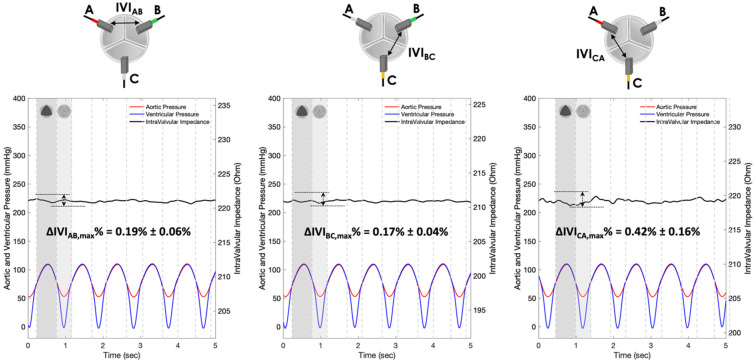
IVI measurements recorded for the three pairs of electrodes of Prototype 2 under normal leaflet dynamics: AB (**left**), BC (**center**) and CA (**right**).

**Figure 12:**
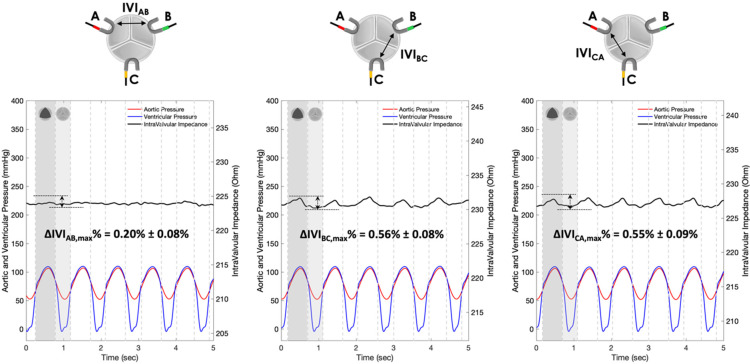
IVI measurements recorded for the three pairs of electrodes of Prototype 3 under normal leaflet dynamics: AB (**left**), BC (**center**) and CA (**right**).

The IVI signals recorded for Prototypes 1, 2 and 3 under normal leaflet dynamics conditions are shown in Figure 10, Figure 11 and Figure 12, respectively.

For each pair of electrodes of Prototypes 1, 2 and 3, the maximum percent variation of the impedance module ($\Delta IVI_{max}\;\%$) was then calculated following Equation (1) (Table 1).

The IVI signal profiles of Prototype 1 (Figure 10) reflect the opening/closing dynamics of the valve leaflets, with maximum values corresponding to complete valve closing and minimum values corresponding to complete valve opening. In particular, the maximum percent variation of the IVI module occurs in the electrode pair BC ($\Delta IVI_{BC,max}\%=3.87\%$), followed by AB ($\Delta IVI_{AB,max}\%=3.20\%$) and CA ($\Delta IVI_{CA,max}\%=2.29\%$)_._

In contrast, the Prototype 2 (Figure 11) do not show an excursion of the impedance variation signal between the opened and the closed states ($\Delta IVI_{AB,max}\%=0.19\%$, $\Delta IV_{BC,max}\%=0.17\%$, $\Delta IVI_{CA},max\%=0.42$), despite the cyclic movements of the valve leaflets within the cardiac cycles.

The IVI signal profiles of Prototype 3 (Figure 10) do reflect, even if with lower excursions than Prototype 1, the opening/closing dynamics of the valve leaflets, with maximum values corresponding to complete valve opening and minimum values corresponding to complete valve closing. In this configuration, the maximum percent variation of the IVI module occurs in the electrode pair BC ($\Delta IVI_{BC,max}\%=0.56\%$) followed by CA ($\Delta IVI_{CA,max}\%=0.55\%$) and AB ($\Delta IVI_{AB,max}\%=0.20\%)$.

### 3.2. Results of Tests with Altered Leaflet Dynamics

**Figure 13:**
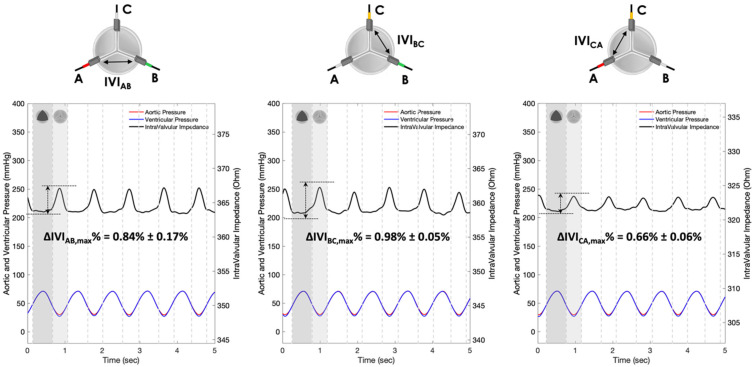
IVI measurements recorded for the three pairs of electrodes of Prototype 1 under altered leaflet dynamics: AB (**left**), BC (**center**) and CA (**right**).

**Figure 14:**
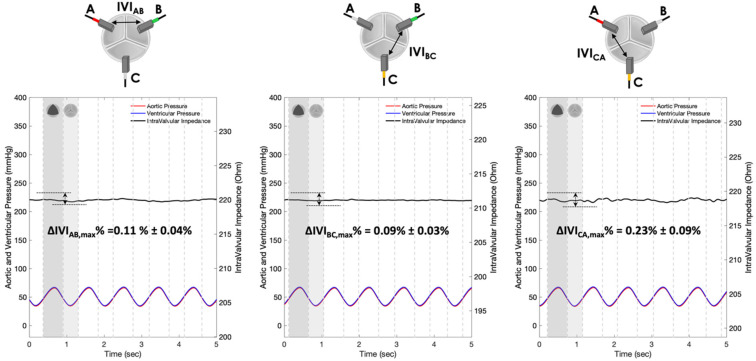
IVI measurements recorded for the three pairs of electrodes of Prototype 2 under altered leaflet dynamics: AB (**left**), BC (**center**) and CA (**right**).

**Figure 15:**
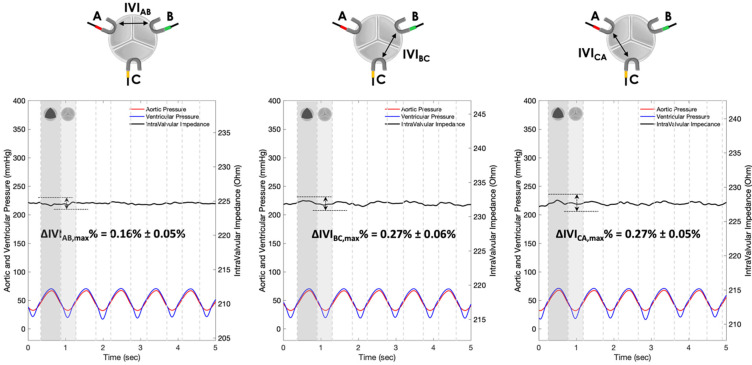
IVI measurements recorded for the three pairs of electrodes of Prototype 3 under altered leaflet dynamics: AB (**left**), BC (**center**) and CA (**right**).

The IVI signals recorded in Prototypes 1, 2 and 3 under altered leaflet dynamics conditions are shown in Figure 13, Figure 14 and Figure 15, respectively.

For each pair of electrodes of Prototypes 1, 2 and 3, the maximum percent variation of the impedance module ($\Delta IVI_{max}\;\%$) was then calculated following Equation (1) (Table 2).

As in the physiological condition, the IVI signal profiles of Prototype 1 (Figure 13) reflect the opening/closing dynamics of the valve leaflets under altered conditions, with maximum values corresponding to complete valve closing and minimum values corresponding to complete valve opening. In particular, the maximum percent variation of the IVI module occurs in the electrode pair BC ($\Delta IVI_{BC,max}\%=0.98\%$), followed by AB ($\Delta IVI_{AB,max}\%=0.84\%$) and CA ($\Delta IVI_{CA,max}\%=0.66\%$)_._

In contrast, and similarly to what obtained in the physiological condition, the IVI signal profiles of Prototype 2 (Figure 14) do not reflect the cyclic movements of the valve leaflets within the cardiac cycles, resulting in low IVI excursions ($\Delta IVI_{AB,max}\%=0.11\%$, $\Delta IV_{BC,max}\%=0.09\%$, $\Delta IVI_{CA,max}\%=0.23)$.

The IVI signal profiles of Prototype 3 (Figure 15) reflect, even if with lower excursions than Prototype 1, the opening/closing dynamics of the valve leaflets, with maximum values corresponding to complete valve opening and minimum values corresponding to complete valve closing. In particular, the maximum percent variation of the IVI module occurs in the electrode pairs BC ($\Delta IVI_{BC,max}\%=0.27\%$) and CA ($\Delta IVI_{CA,max}\%=0.27\%$)_,_ with a lower value in electrode pair AB ($\Delta IVI_{AB,max}\%=0.16\%)$.

We also quantified for each prototype the reduction in the $\Delta IVI_{max}\;\%$ when passing from the “physiological” to “altered” working condition (Table 3). Overall, the Student’s *t*-test revealed a statistically significant reduction in $\Delta IVI_{max}\;\%$ between the “physiological” and “altered” conditions in all the three Prototypes, thereby confirming the sensitivity of IVI measurement to alterations in the valve leaflet movements induced by altered hemodynamics.

## 4. Discussion

In this study we present an innovative intravalvular impedance-sensing concept applied to valve bioprostheses for the monitoring of changes in valve leaflet motion due to the presence of subclinical thrombotic formations.

The hydrodynamic platform allowed us to carry out experimental tests for the assessment of intravalvular impedance (IVI) in different prototypes of sensorized BHVs. By varying the parameters of the circulatory mock-loop platform, it was possible to evaluate a “physiological” working condition which allowed us to simulate the correct leaflet-opening and -closing dynamics, and an “altered” working condition which instead allowed us to simulate the altered leaflet-opening and -closing dynamics which occur in the presence of reduced mobility of the valve leaflets. Even if this model does not exactly simulate the condition of thrombus formation, which would be not easy to experimentally perform, it still reproduces the effect on valve leaflet dynamics that a thrombotic formation would cause, i.e., the reduction in the normal mobility of the leaflets.

The results show that the IVI signal is closely related to the positioning of the electrodes in the valve structure. In the case of electrodes positioned in the commissures (Prototype 1), the maximum IVI signal is when the valve is closed, as the valve leaflets close around the electrodes, maximally interfering with the local electric field lines. By contrast, in the prototypes in which the electrodes were positioned onto the stent along leaflet centerlines (Prototypes 2 and 3), the maximum IVI signal corresponds to the maximum opening of the valve, as the valve leaflets stretch in the direction of the electrodes, maximally interfering with the local electric field lines.

The intra-prototype variability of the IVI measurement, represented by the differences between $IVI_{AB},\;IVI_{BC}\;\mathrm{and}\;IVI_{CA}$ measured in the same prototype, is related to different aspects. First, since it is not possible to carry out simultaneous IVI measurements with the different pairs of electrodes, the signal recorded with each pair refers to a different heartbeat, which by its nature has an intrinsic variability. Second, the valve prostheses do not have perfectly symmetrical leaflets. This heterogeneity in their geometry determines different opening and closing dynamics for each leaflet, thus introducing a variability in the measured IVI signal between each pair of electrodes and, consequently, also of the $\Delta IVI_{max}\%$ values calculated following Equation (1). Third, the measuring electrodes positioned inside the valve structure may have variable spatial orientations and/or inclinations due to the difficulties in their positioning which, being manually executed by the operator, does not ensure a perfectly symmetric positioning inside the valve structure. Fourth, even starting from electrodes of the same shape and size, the effective surface depends on the portion of electrode covered by the heat shrink (applied manually) at the electrode-wire interface, which results in different IVI measurements at the baseline as described by Ohm’s second law.

By improving the manufacturing and embedding of the sensing electrodes in the prosthesis, much of this variability may be reduced and controlled. In this way, the remaining detected variability of the IVI signal from one pair of electrodes to another may be attributable to an altered motion of the leaflet monitored by the specific pair of electrodes, thus implying a possible altered condition that would be interesting to detect.

Experimental tests carried out in vitro under “physiological” working conditions have shown greater sensitivity to IVI measurement by Prototype 1 (Figure 10), which involves “mini-platelet” electrodes inserted in the valve commissures. In such a prototype, the maximum excursion of the impedance-variation signal between the opened and the closed valve condition has been identified. By contrast, in Prototype 2 (Figure 11), in which the measuring “bar” electrodes were placed onto the valve stent, it was not possible to identify any cyclic impedance-variation signal reflecting the valve dynamics, probably due to the leaflets not getting close enough to the electrodes. In Prototype 3 (Figure 12), characterized by the same electrode positioning as Prototype 2 but with “arch” electrodes, it was possible to detect a small IVI excursion during opening/closing dynamics, but only in two pairs of electrodes (BC and CA), and albeit to a lesser extent than in Prototype 1. This excursion, however, does seem to depend on the presence of one of the leaflets, more precisely the one facing electrode C, which comes into contact with the measuring electrode during the opening phase, thus determining the peak of the IVI signal. This justifies why there is no IVI excursion within the cardiac cycle in the electrode pair AB, as it does not involve electrode C.

The experimental tests carried out in vitro under “altered” working conditions (Figure 13, Figure 14 and Figure 15) have shown, in all three Prototypes, a reduction in the maximum percent variation of the impedance module ($\Delta IVI_{max}\;\%$) in comparison with the values obtained in the “physiological” working condition. The quantification of the reduction in the $\Delta IVI_{max}\;\%$ values when passing from the “physiological” to “altered” working condition is reported in Table 3 as a percentage. The comparison between the three Prototypes reveals that the Prototype 1 shows the highest $\Delta IVI_{max}\;\%$ reduction (74.6%), followed by Prototype 3 (52.7%) and Prototype 2 (45.4%). As a result, the electrode configuration provided by Prototype 1, which is characterized by “mini-platelet” electrodes embedded in the commissures of the valve leaflets, seems to be the optimal electrode configuration to carry out the IVI measurement. Moreover, the “mini-platelet” electrodes positioned into the valve commissures may represent the least intrusive sensing solution for the bioprosthesis, and the most versatile option, as it is potentially applicable to several models of valve prostheses that do not necessarily have an external stent to which the electrodes can be anchored.

### Study Limitations and Future Directions

Study results are limited to in vitro tests of proof-of-concept prototypes of sensorized valve bioprostheses inserted inside a benchtop simulator that does not allow us to reproduce the real in vivo environment and, as such, can influence the measured IVI signal. The main limitations of the preceding study are related to the use of a working fluid which allows us to simulate the electrical conductivity of the blood [26] but not its viscosity, and the absence of a flow sensor inside the Pulse Duplicator for the monitoring of transvalvular flow during the acquisitions. Further ex vivo experimental trials and in vivo animal experiments will be needed to confirm these preliminary observations.

**Figure 16:**
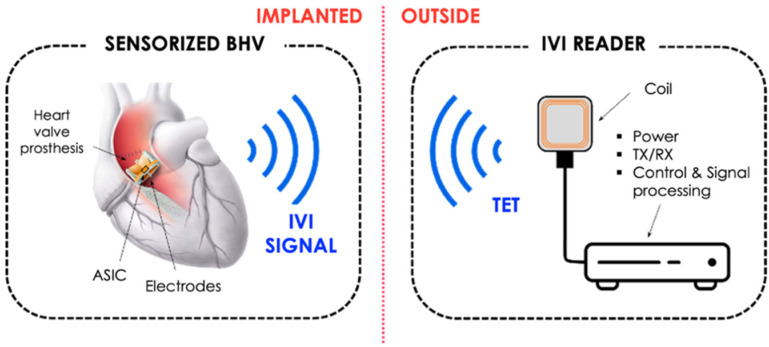
Overall design for an implantable sensorized BHV for IVI sensing.

Further studies will also be necessary to create an implantable prototype of the sensorized prosthesis by removing the current wired connections. An example scheme of the final sensorized device is shown in Figure 16.

The sensorized BHV for IVI sensing will be equipped with a miniaturized Application-Specific Integrated Circuit (ASIC) and means for telemetric communication with the external electronic measurement unit (IVI reader) which powers and interrogates the implanted sensor system. The impedance measurement unit, which is currently represented by the external impedance-meter should be integrated within the ASIC. Therefore, the ASIC should include a current pulse generator, an electrode connection unit, a signal conditioning unit (amplification and filtering) and a TX-RX unit for signals transmission and receiving. The power supply of the implanted device will be guaranteed by the insertion of a dedicated coil for Transcutaneous Energy Transmission (TET) by inductive coupling with the external IVI reader. Surely, the location of both ASIC chip and micro coil for inductive coupling represents a critical point. Indeed, they should be located in a proper site of the valve prosthesis so as not to hinder in any way the natural movement of the valve leaflet. In this sense, a possible location to investigate for future development is the prosthesis valve ring.

Furthermore, different biomaterials and manufacturing techniques for the realization of the electrodes in the final implantable prototype will be evaluated to guarantee their biocompatibility and long-term durability.

## 5. Conclusions

In this study we presented the conceptual design of IVI sensing applied to a commercial BHV model. Different solutions for the sensorization of the BHVs were presented and tested on a circulatory mock-loop platform reproducing “physiological” and “altered” leaflet dynamics. Overall, the results of our investigations confirmed a sensitivity of the IVI measurement to experimentally induced changes in valve leaflet motions, specifically for the sensorized BHV prototype which involved small parallelepiped-shaped electrodes embedded in the commissures of the BHV leaflets. Thus, our results are promising for further developing the IVI sensing approach, which may be able to detect the presence of subclinical thrombotic formations early after prosthesis implantation and monitor the progressive deterioration of the prosthesis itself.

Further studies will be needed to achieve an implantable solution of an IVI-sensorized BHV, and for ex vivo and in vivo animal experimentations.

## 6. Patents

From the work reported in this manuscript, the following issued patents result:

- WO2015EP58201 20150415. Heart valve prosthesis with integrated electronic circuit for measuring intravalvular electrical impedance, and system for monitoring functionality of the prosthesis. E. Marcelli (Inventor); Alma Mater Studiorum (Applicant). Filed: 15 April 2015.
- Also published as: EP3131502 (A1); CN106456043 (A); US9987129 (B2)—Issued: 5 June 2018.
- N. 0001423344 Protesi valvolare cardiaca con circuito elettronico integrato per effettuare misure di impedenza elettrica intravalvolare e sistema per monitorare la funzionalità di tale protesi—E. Marcelli (Inventor); Alma Mater Studiorum (Applicant). Filed: 16 April 2014. Issued: 22 July 2016.

## Figures and Tables

**Table 1:** For each pair of electrodes (AB, BC and CA) of the three prototypes, maximum percent variation of the impedance module ($\Delta IVI_{max}\%$), reported as Mean Value±SD, is shown for the “physiological” working condition.

Prototypes	$\Delta IVI_{AB,max}\%\pm SD$	$\Delta IVI_{BC,max}\%\pm SD$	$\Delta IVI_{CA,max}\%\pm SD$
Prototype 1	3.20%±0.09%	3.87%±0.14%	2.29%±0.07%
Prototype 2	0.19%±0.06%	0.17%±0.04%	0.42%±0.16%
Prototype 3	0.20%±0.08%	0.56%±0.08%	0.55%±0.09%

**Table 2:** For each pair of electrodes (AB, BC and CA) of the three prototypes, maximum percent variation of the impedance module ($\Delta IVI_{max}\%$), reported as Mean Value±SD, is shown for the “altered” working condition.

Prototypes	$\Delta IVI_{AB,max}\%\pm SD$	$\Delta IVI_{BC,max}\%\pm SD$	$\Delta IVI_{CA,max}\%\pm SD$
Prototype 1	0.84%±0.17%	0.98%±0.05%	0.66%±0.06%
Prototype 2	0.11%±0.04%	0.09%±0.03%	0.23%±0.09%
Prototype 3	0.16%±0.05%	0.27%±0.06%	0.27%±0.05%

**Table 3:** Summary of $\Delta IVI_{max}\%$ values obtained in both “physiologic” and “altered” working conditions in the three Prototypes, with the respective value of $\Delta IVI_{max}\%$ reduction (given as %) when passing from “physiological” to “altered” working conditions. *p*-value obtained with Student’s *t*-test is reported.

	Electrodes	$\Delta IVI_{max}\%$ “Physiologic” Condition”	$\Delta IVI_{max}\%$ “Altered” Condition	Reduction %	*p*-Value
Prototype 1	AB	3.20%	0.84%	73.6%	<0.01
	BC	3.87%	0.98%	74.6%	<0.01
	CA	2.29%	0.66%	71.2%	<0.01
Prototype 2	AB	0.19%	0.11%	40.8%	<0.01
	BC	0.17%	0.09%	43.3%	<0.01
	CA	0.42%	0.23%	45.4%	<0.01
Prototype 3	AB	0.20%	0.16%	18.1%	=0.04
	BC	0.56%	0.27%	52.7%	<0.01
	CA	0.55%	0.27%	51.2%	<0.01

## Data Availability

The datasets generated during and/or analyzed during the current study are available from the corresponding author on reasonable request.
